# A Fully Implantable Pacemaker for the Mouse: From Battery to Wireless Power

**DOI:** 10.1371/journal.pone.0076291

**Published:** 2013-10-23

**Authors:** Jacob I. Laughner, Scott B. Marrus, Erik R. Zellmer, Carla J. Weinheimer, Matthew R. MacEwan, Sophia X. Cui, Jeanne M. Nerbonne, Igor R. Efimov

**Affiliations:** 1 Department of Biomedical Engineering, Washington University in Saint Louis, Saint Louis, Missouri, United States of America; 2 Department of Internal Medicine, Division of Cardiovascular Sciences, Washington University in Saint Louis, Saint Louis, Missouri, United States of America; 3 Department of Developmental Biology, Washington University in Saint Louis, Saint Louis, Missouri, United States of America; University of Minnesota, United States of America

## Abstract

Animal models have become a popular platform for the investigation of the molecular and systemic mechanisms of pathological cardiovascular physiology. Chronic pacing studies with implantable pacemakers in large animals have led to useful models of heart failure and atrial fibrillation. Unfortunately, molecular and genetic studies in these large animal models are often prohibitively expensive or not available. Conversely, the mouse is an excellent species for studying molecular mechanisms of cardiovascular disease through genetic engineering. However, the large size of available pacemakers does not lend itself to chronic pacing in mice. Here, we present the design for a novel, fully implantable wireless-powered pacemaker for mice capable of long-term (>30 days) pacing. This design is compared to a traditional battery-powered pacemaker to demonstrate critical advantages achieved through wireless inductive power transfer and control. Battery-powered and wireless-powered pacemakers were fabricated from standard electronic components in our laboratory. Mice (n = 24) were implanted with endocardial, battery-powered devices (n = 14) and epicardial, wireless-powered devices (n = 10). Wireless-powered devices were associated with reduced implant mortality and more reliable device function compared to battery-powered devices. Eight of 14 (57.1%) mice implanted with battery-powered pacemakers died following device implantation compared to 1 of 10 (10%) mice implanted with wireless-powered pacemakers. Moreover, device function was achieved for 30 days with the wireless-powered device compared to 6 days with the battery-powered device. The wireless-powered pacemaker system presented herein will allow electrophysiology studies in numerous genetically engineered mouse models as well as rapid pacing-induced heart failure and atrial arrhythmia in mice.

## Introduction

The use of animal models of human disease processes plays an essential role in bio-medical research. Models of cardiovascular disease offer unique challenges since these diseases are multifactorial, often with contributing environmental causes that take years to fully manifest. One versatile maneuver is altering heart rate with the implantation of a cardiac pacemaker, a small electronic device connected to the heart with wires or leads. In animal models, the induction of rapid heart rates over a 4–6 week period results in cardiac remodeling and dysfunction, providing a model system of high impact cardiac diseases including congestive heart failure [1] and atrial fibrillation [2]. In addition, even at normal heart rates, it has been shown that long-term right ventricular pacing in humans can have deleterious effects on cardiac function, an observation which has led to altered clinical guidelines for pacemaker programming [3].

Although pacemakers were originally and primarily designed as a treatment for slow heart rate (bradyarrhythmias), recent developments have begun to highlight an array of more complex and subtle effects of ectopic pacing on cardiac electrical function. One seemingly simple example of pacemaker induced remodeling is the observation that after a period of ectopic pacing, a persistent alteration in T-wave axis (observed on the body surface ECG) occurs, which can linger for weeks or months [4]. Although originally viewed as a clinical curiosity, increasing research indicates that this phenomenon known as cardiac memory provides insight into the cellular and molecular changes provoked by pacing [5]. A tentative model of pacemaker-induced changes includes local angiotensin signaling, leading to ion channel remodeling and resulting in heterogeneous prolongation of action potentials. However, a complete description of ion channel remodeling in cardiac memory is lacking and the underlying molecular mechanisms remain incompletely understood [6].

One challenging aspect of studying pacemaker-induced phenomenon is the choice of model system. Traditionally, larger animal models, primarily canine, have been used to allow easier implantation of the pacemaker. On the other hand, the mouse, with its long history as a versatile and affordable genetic and molecular model, offers a much wider range of tools to investigate electrical remodeling in models of heart failure on the gene/protein levels [7]. The first general feasibility study of pacemaker implantation in the mouse was illustrated in a study by Bilchick et al. in which they demonstrated that epicardial right ventricular (RV) pacing in the mouse resulted in mechanical dyssynchrony and differential gene expression in the lateral versus the septal walls of the LV [8]. While Bilchick et al. were able to document early signs of pacemaker-induced remodeling in the mouse, their pacemaker only permitted reliable chronic pacing for a maximum of 1 week at a rate of 720 BPM. The current study was undertaken to design a pacemaker capable of pacing the mouse heart for 3–4 weeks in order to permit further exploration of pacemaker-induced remodeling in the mouse. Here, we describe the development of a fully implantable mouse pacemaker from a traditional battery-powered design to a novel, low-cost, wireless-powered pacemaker platform capable of pacing up to 30 days.

## Methods

### Fabrication of Battery-powered Pacemaker

**Figure 1:**
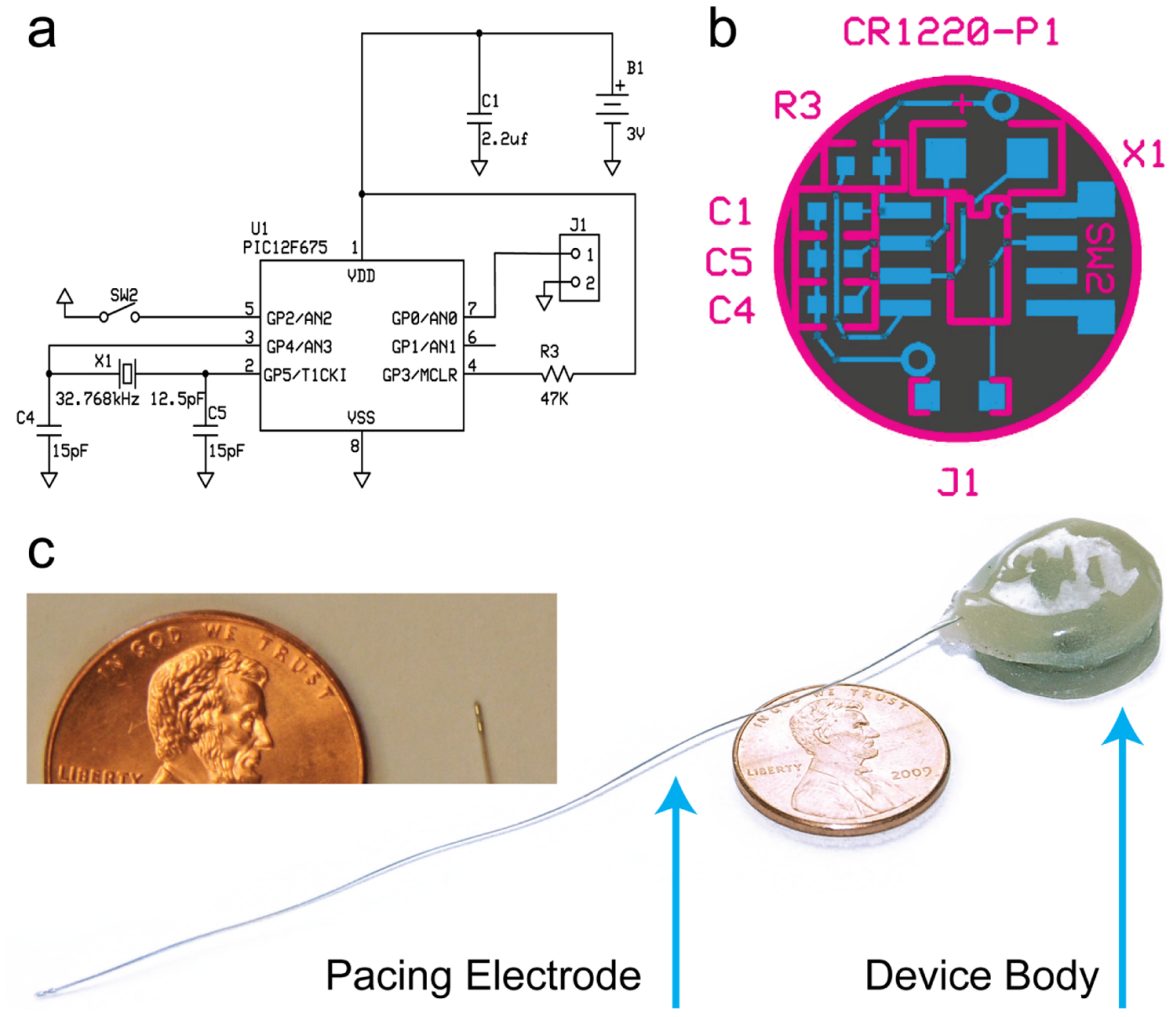
Layout of the battery-powered mouse pacemaker. (a) Circuit design. (b) Printed circuit board. (c) Battery-powered pacemaker coated in biocompatible epoxy with endocardial pacing catheter attached. Inset image features the tip of the bipolar pacing catheter.

We designed our printed circuit board (PCB) layout and pacemaker circuit using free ExpressPCB CAD software (expresspcb.com). Our PCB (**Figure 1a**) is a two-layer board with a bottom copper layer (gray) for grounding and top copper web (blue) for component attachment. Individual circular PCBs were extracted from a card of 12 chips. All surface mount components were manually soldered to each chip and can be ordered through Digi-Key Corp. (Thief River Falls, MN) or Mouser Electronics Inc. (Mansfield, TX). Prior to attachment, the PIC microcontroller is reprogrammed with our source code using the PICkit 2 Development Programmer (Microchip Technology Inc., Chandler, AZ), and 8-SOIC to 8-DIP adapter, and Microchip’s MPLAB software. Source code for the microcontroller can be found the Online Appendix.

### Implant Procedure for Battery- and Wireless-powered Pacemakers

All animal studies were carried out in strict accordance with the recommendations in the Guide for the Care and Use of Laboratory Animals of the National Institutes of Health. The following protocol was approved by the Institutional Animal Studies Care and Use Committee of Washington University in St. Louis (Protocol Number: 2010005 and 2013001). Adult mice (n = 14) were anesthetized with ketamine (100 mg/kg) and xylazine (10 mg/kg) administered intraperitoneally and then restrained in a supine position on a heated magnetic stainless steel surgical board under a low power binocular dissecting microscope. In our original attempts to surgically implant a battery-powered pacemaker, we used an external jugular approach. For this procedure, mice were prepared for surgery with aseptic and anesthetic techniques. The mice were intubated with a 20-gauge 1″ smooth needle through the oral cavity as verified by direct visualization of the intubation device through the exposed trachea. Mice were then ventilated at a tidal volume of 200–500 µl and a rate of 140 strokes/minute. After mice reached an anesthetic level for surgery, the neck skin was cut to expose the jugular vein. The vein was isolated and secured with suture. A sterile battery-powered pacemaker was then inserted into a subcutaneous pocket formed by blunt dissection along the animal’s flank and back. The pacing electrode was then fed through a sterile trocar under the skin and into the site of the jugular isolation. A small incision was made in the jugular vessel and the pacemaker lead was inserted through the vessel and into the right ventricle of the heart. After confirmation of pacing capture and good positioning of the extra wire length, the pacemaker lead was secured into the jugular by tying down the suture and placing a small drop of sterile tissue glue on the vessel at the site of lead entrance. Both the neck and back skin incisions were closed and mice were allowed to recover on a warmer until being returned to their cage.

For the wireless-powered pacemaker, aseptic and anesthetic techniques were followed as previously described in adult mice (n = 10). Once a surgical plane of anesthesia was achieved in the mouse, a small incision was made in the mid-thorax region followed by blunt dissection to expose the larynx and trachea. A short midline skin incision was made with reflection of the skin directly above the base of the sternum. A subcutaneous skin pocket was made below the sternum and right above the abdomen by blunt dissection for placement of the flat pacemaker device. The pacemaker was then positioned under the skin in this pocket to meet the exact length required for the electrodes to enter the chest at the 4^th^ intercostal space. The chest was then opened by a lateral cut along the left side of the sternum between the ribs. The chest wall was retracted to better expose the apex of the left ventricle. The sterile pacemaker electrode was then sewn with 8–0 suture to the apical myocardial wall through either the anodal or cathodal eyelet. It was also ensured that both electrodes were touching the surface of the ventricular wall. The chest wall was then closed around the electrode with a purse string. The surgical incision was closed in two layers with an interrupted suture pattern. Mice were then allowed to recover with oxygen on a heating pad until extubation. Mice were monitored closely for 3 days following surgery.

Following implantation and recovery, mice were monitored daily. In cases of erosion of the device through the skin, mice were euthanized at the time the erosion was identified. In all other cases of death, no symptoms were evident prior to death. At the time of sacrifice, mice were anesthetized with Avertin (0.5 mg/g) followed by removal of the heart for inspection and identification of complications.

### Statistical Analysis

All statistical analysis was performed in Prism (GraphPad Software, Inc., La Jolla, CA). An F-test was performed on the slope of the regression line of the mean pacing pulse width thresholds to determine significance compared to a slope of 0.

## Results

### Battery-powered Pacemaker

We began by creating a battery-powered design similar to Bilchick et al. in order to better understand design criteria for an implantable mouse pacemaker (**Figure 1**). Our battery-powered device is based on the PIC12F675 microcontroller (Microchip Technology Inc., Chandler, AZ). We chose the PIC12F675 for its small size, low-cost and internal memory that enables 14 different programmable stages. Using this microcontroller, we produced a pacing circuit (**Figure 1a**) powered by a 3 V lithium coin-cell battery (CR1220, 38 mAh). Additionally, we employed an external 32 kHz crystal oscillator for low-power timing of stimulation rates and pulse widths. Our design features 14 unique combinations of pulse width (1 ms, 1.5 ms, 2.0 ms, or 2.5 ms) and pacing rate (600 PPM, 700 PPM, 800 PPM, or 1000 PPM) on a circular buffer, a low-power hibernation mode, and a magnetic reed switch for external control once implanted. All circuit elements were attached to a custom-designed, two-layer PCB (**Figure 1b**). One key component of the pacemaker from Bilchick and colleagues was an epicardial pacing electrode with a fixation screw produced by Boston Scientific (St. Paul, MN; formerly Guidant Corporation). Since this lead was not available to us, we substituted a 1 Fr bipolar murine pacing catheter produced by NuMed Inc. (Hopkinton, NY) on our device (**Figure 1c**). Similar to human pacemaker implantation procedure, this pacing catheter was guided into the right ventricle via the right jugular vein of the mouse and used for endocardial pacing. To protect the device against degradation from biological fluids, each device is embedded in EPO-TEK 730 general-purpose epoxy (Epoxy Technology, Inc., Billerica, MA). Fully assembled devices measure 17.20±0.23 mm (diameter)×8.88±0.63 mm (thickness) with a mass of 3.07±0.10 g.

**Figure 2:**
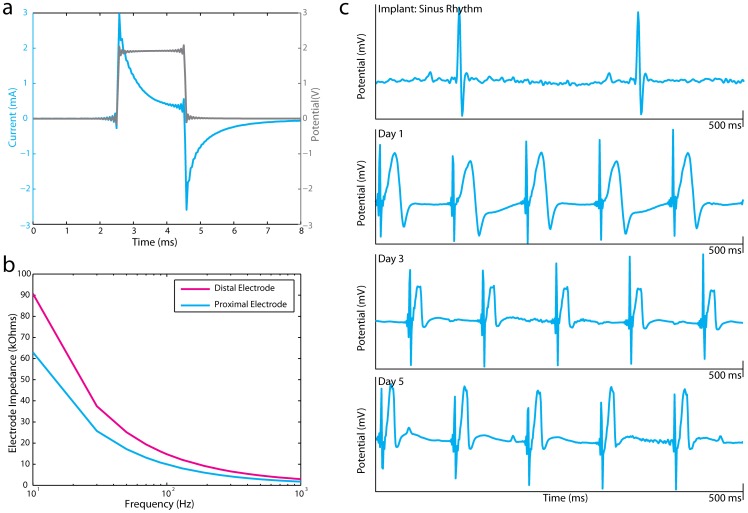
Bench top and *in vivo* testing of battery powered pacemaker. (a) Current (blue) and voltage (gray) traces from the pacing catheter. (b) Frequency Response Analysis of distal and proximal pacing catheter electrodes (c) Lead II ECG recording in a mouse heart during sinus rhythm and right ventricular pacing by the battery-powered pacemaker over 5 days.

Similar to human pacemakers, this battery-powered mouse pacemaker operates as a constant voltage pulse generator. **Figure 2a** displays current and voltage traces recorded from the pacing catheter in a saline bath. Additionally, we performed a Frequency Response Analysis (FRA) on the pacing catheter to measure electrode impedance as a function of waveform frequency content (**Figure 2b**). Lead impedance of the pacing catheter varies from 2.98–90.74 kΩ on the distal electrode and 1.78–62.99 kΩ on the proximal electrode. Based on bench-top testing and battery rating (38 mAh), we estimated that this device should pace reliably for approximately 30 days.

Battery-powered devices were implanted in mice (n = 14) as specified in the *Methods* section. Following initial implantation, device functionality was tested via an electrocardiogram (ECG) and an echocardiogram to verify electrical and mechanical cardiac capture. Mice were monitored daily for survival and device functionality via an ECG. All ECG measurements were performed under inhaled isofluorane anesthesia (1–2%). Eight of 14 (57.1%) mice died within 1–2 days of implantation due to various causes (e.g. RV perforation, surgical trauma, stroke, device removal by animal). Of the survivors, 2 of 6 (33.3%) survived 4 days, 1 of 6 (16.7%) survived 6 days, 1 of 6 (16.7%) survived 8 days, and 2 of 6 (33.3%) survived 11 days. With respect to device functionality, pacing was achieved in 9 of 14 (64.3%) mice. Chronic pacing was achieved in two of the 4 (50%) mice that survived >4 days for a maximum of 5 days and 6 days. **Figure 2c** depicts Lead II ECGs recorded during sinus rhythm and chronic RV pacing over a 5-day period.

### Wireless-powered Pacemaker

**Figure 3:**
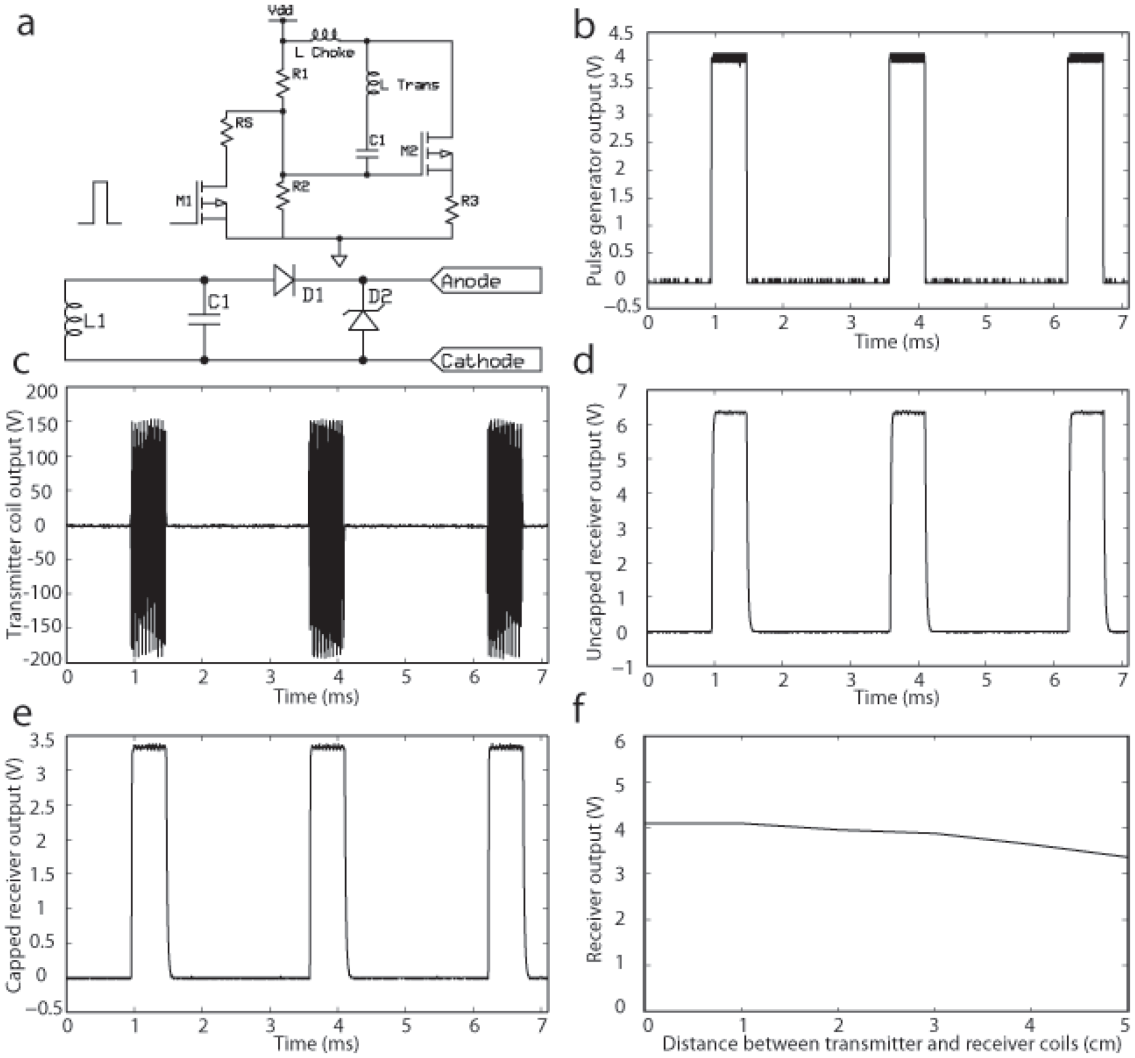
Layout of the wireless-powered pacemaker. (a) Circuit layout of transmitter (top) and receiver (bottom). (b) Pulsed input into transmitter from pulse generator. (c) Output from transmitter. (d) Uncapped output from receiver. (e) Capped output from receiver. (f) Receiver output decreases minimally up to 5 cm from the transmitter coil. See text for further details.

Due to the high post-implant mortality with the battery-powered pacemaker, we attempted to refine our pacemaker design. We hypothesized that implant intolerance was primarily due to device size as well as mechanical complications resulting from placement of the endocardial lead. One of the larger components of the battery-powered design, by mass and volume, is the battery. Presently, lithium ion batteries with smaller size and sufficient charge capacity do not exist. As a result, we began exploring wireless energy transfer as a means for device power without the use of a battery. Inductive energy transfer has been used extensively for both power and data communication in implantable devices for various biological applications [9]–[11]. Similar to these designs, we developed a wireless-powered pacemaker consisting of three major components: a function generator, an external transmitter, and a passive receiver. Power transmission is achieved through a modified Class-E power oscillator (**Figure 3a** **, top**) (EZ, MM, JL; Unpublished data). An external function generator (26T, AD Instruments, Inc., Colorado Springs, CO) is used to generate a pulsed input (**Fig. 3b**) that modulates pacing rate and pulse width of an external transmitter circuit. This oscillator circuit operates at a carrier frequency of 5 MHz and approximately 390 V peak-to-peak (**Figure 3c**). Near-field magnetic coupling occurs between an external transmitter coil and a tuned, implanted receiver circuit that drives constant-voltage, monophasic pulses through the implant (**Fig. 3a** **, bottom**). As a result of the large voltage from the transmitter, we were able to design a passive receiver without additional amplifiers. **Figure 3d** displays the output of the receiver over a 5-kΩ resistor with a coil-to-coil distance of 45 mm. Since the minimal cage size required for housing a single mouse is 4 cm×4 cm, this range is adequate to permit powering a device in a freely moving mouse. However, additional optimization of the transmitter/receiver system can be made in the future to increase this range. To reduce the influence of mouse position within the transmitter coil, a Zener diode was connected to the output of the device to cap the stimulation voltage at 3.9 V. **Figure 3e** demonstrates the output waveform after diode capping. **Figure 3f** demonstrates that device output remains relatively constant over a range of 0–5 cm from the transmitter; this output was sufficient at 5 cm for reliable cardiac pacing. All electrical components for the transmitter and receiver are listed in **Table 1**.

**Table 1:** Parts list for wireless transmitter and receiver circuits.

Transmitter Circuit	
Part Symbol	Part Value
M1	TN5325K1-G
M2	IRFSL5620PBF-ND
R1/R2	10 kΩ
R3	18 Ω
RS	330 Ω
L Trans	9.1 µH
L Choke	150 µH
C1	138 pF
**Receiver Circuit**	
**Part Symbol**	**Part Value**
D1	CDBU0130L
D2	CZRUR52C3V9
C1	176 pF
L1	0.79 µH

**Figure 4:**
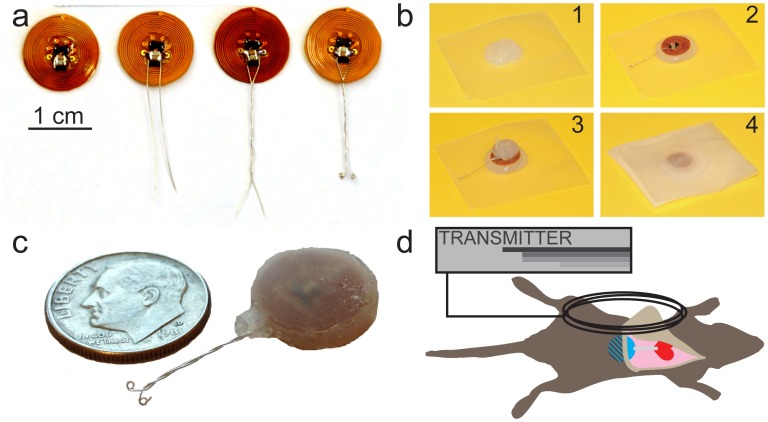
Assembly process of wireless powered pacemaker. (a) Platinum wire is attached to the circuit board, wound together, and coiled with a 0.5 cc syringe. (b) A bead of Silastic is placed on a piece of parafilm(1). The device is placed on the bead(2), coated with an additional layer of Silastic(3), and topped with a piece of gas permeable film(4). (c) Final product. (d) Artistic rendering of external transmitter interacting with abdominally implanted receiver in mouse.

The receiver coil and pacing circuit are assembled on a flexible polyimide PCB (Red Rock Laboratories, LLC, St. Louis, MO) to reduce device thickness and attached to a platinum bipolar pacing electrode. **Figure 4a** demonstrates the lead attachment process to the polyimide PCB. Briefly, two pieces of PFA-insulated platinum-iridium wire (0.005″ bare uncoated, 0.008″ coated, A-M Systems, Sequim, WA) are soldered to the PCB. The wires are braided together and coiled at the ends with a 0.5cc syringe. Several iterations of the pacing electrode were attempted (straight wire, braided wire, and braided wired with eyelets) before settling on the pacing electrode in the final design (**Figure 4c**). We found that the braided wire with eyelets sutured to the apex minimized the potential for serious complications due to an internal puncture wound. Once the pacing electrode is attached, the implant is embedded in Silastic medical adhesive (Dow Corning, Midland, MI) (**Figure 4b**). Silastic was selected for device potting to reduce the mechanical mismatch between the lead and the circuit board, minimizing potential shearing of the lead. Device encapsulation is performed by first depositing a bead of Silastic on parafilm (**Figure 4b** **1**). Next, the device is pressed into the bead of Silastic (**Figure 4b** **2**) and an additional layer is added to the top (**Figure 4b** **3**). A final layer of areaseal film is pressed on the top of the device to join the upper and lower layers of Silastic (**Figure 4b** **4**). The use of gas-permeable areaseal film was found to be essential to allow curing of Silastic. The device is cured at room temperature for 3 days and then trimmed of excess. **Figure 4c** shows the final assembled product. Each device measures approximately 13.65±0.25 mm (diameter)×3.27±0.50 mm (thickness) and weights 0.56±0.07 g, a 82% reduction by mass and 77% reduction by volume compared to the battery-powered design. In order to reduce surgical complexity, our wireless design was developed as an epicardial pacemaker. Briefly, the receiver is placed subcutaneously in the mouse abdominal region and the pacing lead is sutured to the epicardium of left ventricular (LV) apex. A more complete description of the implant technique is provided in the *Methods* section.

Wireless-powered devices were implanted in mice (n = 10) as specified in the *Methods* section. Device functionality was verified in all ten (100%) mice at implant via ECG. Echocardiographic measurements were not acquired due to interference concerns between the transmitter and the echo machine. Following a five-day recovery period, ventricular pacing was conducted under isofluorane anesthesia (1–2%) at 5 day intervals for 30 days. Due to the fact that a cage system incorporating the transmitter coils remains under development, the wireless device was not continuously powered (in contrast to the battery device described above) but was only powered with a transmitter coil during testing at 5 day intervals to verify stability of device function. At each follow-up, pacing pulse-width threshold at 600–630 BPM was tested. On post-operative day 30, ability to pace above intrinsic physiologic rates (>750 BPM) was tested.

**Figure 5:**
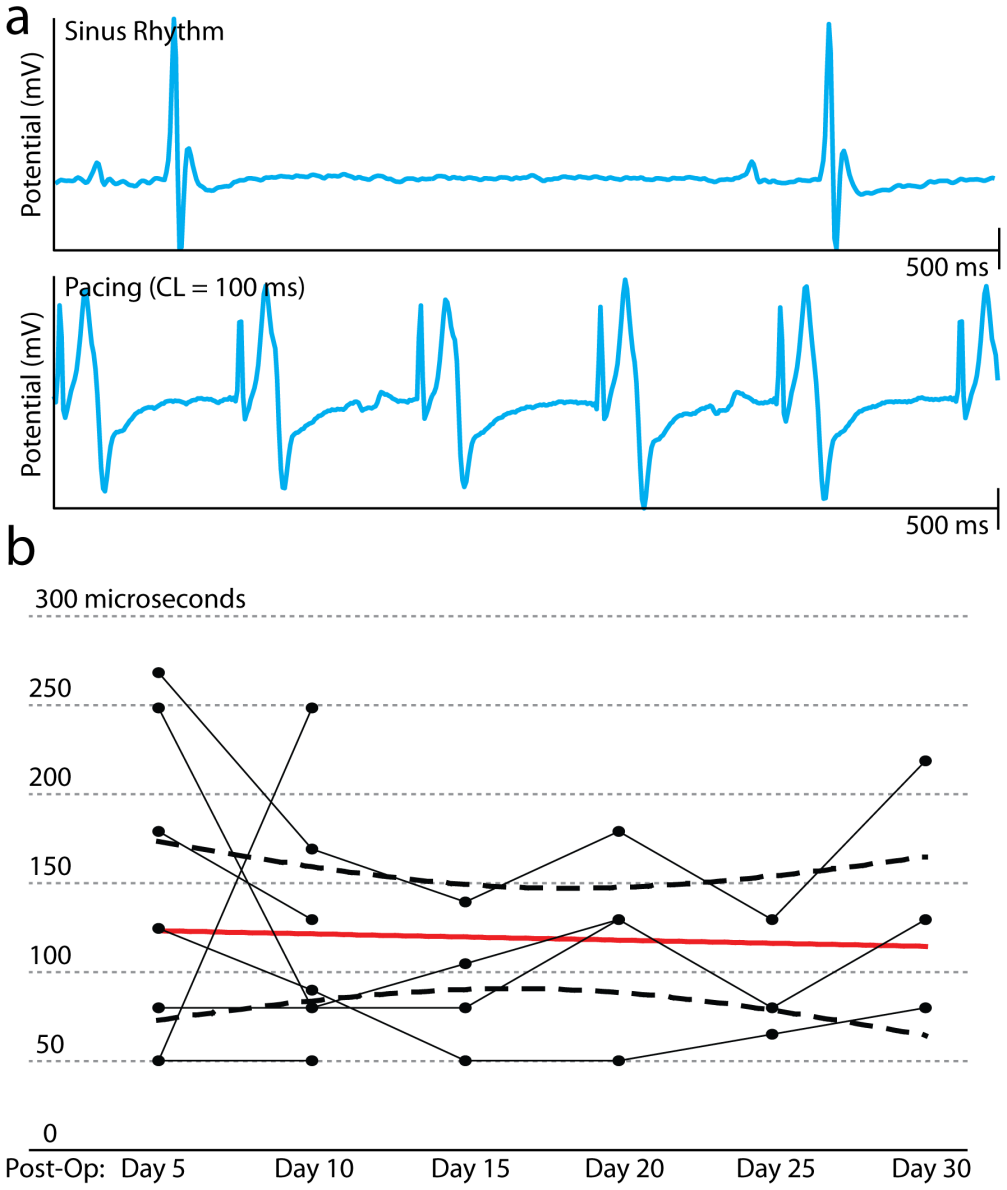
*In vivo* testing of wireless pacemaker. (a) Lead II ECG during normal sinus rhythm (top) and during LV apical pacing (bottom). (b) Pacing pulse width threshold of wireless device over 30 days for all mice with stable capture. Solid red line shows linear regression on mean pulse width thresholds. Dashed black lines show 95% confidence interval bounds for the regression.

Nine of 10 (90%) mice survived device implantation with the wireless-powered device. Of the survivors, 6 of 9 (66.7%) mice survived 20 days (one mouse died on postoperative day 5, one on postoperative day 14, and one was euthanized on postoperative day 10 due to erosion of the device through the skin). Necropsies were performed but no cause of death was identified. Twenty days post implant, 5 of 6 devices functioned properly. One device exhibited unreliable capture at 600 BPM; therefore pacing with reliable cardiac capture was successfully achieved in 4 of 6 (66.7%) mice for 20 days. In addition, three devices continued to exhibit stable function for up to 30 days. **Figure 5a** shows a representative example of normal sinus rhythm and pacing at 600 BPM. Pacing at >750 BPM was achieved in all three (100%) mice with functional devices on post-operative day 30. **Figure 5b** shows the measured pulse width thresholds over the 30-day period for all mice with stable device function. An ordinary least squares regression on the mean pulse width threshold with 95% confidence interval bounds is superimposed in red (slope = −0.1±1.1 µs/day, p = 0.93 compared to a slope of 0).

## Discussion

This study describes the design and implementation of a miniature, implantable wireless pacemaker for mice with off-the-shelf electronic components. This wireless design described herein has considerable advantages over a traditional battery-powered pacemaker. A list of contrasting device features is provided in **Table 2**.

**Table 2:** Comparison of battery-powered and wireless-powered pacemakers.

DEVICE	Battery	Wireless
**MASS**	3.07 g	0.56 g
**SIZE**	8.9×17.2 mm	3.3×13.7 mm
**LIFE**	30 days	∼3 month
**CONTROL**	microchip	external
**SETTINGS**	15 stages	infinite
**COST**	$275	$30

First and foremost, our wireless-powered pacemaker is 82% smaller by mass and 77% smaller by volume compared to our battery-powered design. Compared to the battery-powered device described by Bilchick et al. [8], our wireless device is 66.7% smaller by mass. Reduction in the amount of implantable material is likely a key factor to reducing the morbidity and mortality of the pacemaker implant procedure. Despite our best efforts, a significant incidence of animal mortality was observed immediately following implantation of our battery-powered device: 57.1% with the battery-powered pacemaker (similar to a 40% mortality rate reported by Bilchik et al.) compared to 10% with the wireless-powered pacemaker. High implant mortality may be largely attributed to the large size of the battery-powered devices in our design and that of Bilchick and colleagues.

Second, we demonstrate successful pacing for 30 days with our wireless device, a significant improvement compared to the one week of reliable pacing reported in previous studies [8]. This increase in device life can be attributed to the use of wireless power in our design. Our wireless device is not limited by finite battery life or rapid battery drain due shorting of the device once implanted. In bench-top testing in a saline bath, pacemaker function remained stable for 3 months.

External control of our wireless device increases device flexibility and functionality. Pulse width and pacing rate can be adjusted on demand and are not limited to values preprogrammed onto a microchip. Additionally, programmed stimulation protocols (e.g., S1S1, S1S2, S1S3, etc.) can be created on an external function generator to perform sophisticated electrophysiological testing *in vivo*.

Finally, cost and ease of implant were carefully considered during the development of a fully implantable pacemaker for mice. In our original battery-powered design, we used a 1 Fr. murine pacing catheter developed by NuMed, Inc. as our pacing electrode. While the placement of this electrode in the RV endocardium more closely resembles pacing in canines, consistent placement of the pacing catheter in the RV required a skilled mouse surgeon and occasional use of echocardiography and, even so, increased peri-operative mortality due to RV perforation. Additionally, the NuMed catheter added significant cost to the battery-powered design ($250 of the $275 total cost). By decreasing device size and pursuing an epicardial pacing strategy, we were able to greatly reduce the complexity of the implant procedure. Moreover, by replacing the NuMed catheter with standard platinum-iridium wire, a complete build for a wireless-powered pacemaker can be assembled for approximately $30.

## Conclusions

Here we demonstrate a successful implementation of a fully implantable mouse pacemaker based on wireless power transfer and control capable of 30 days of in vivo pacing. For the purpose of device testing, all experiments in this study were performed on anesthetized animals with a hand-held transmitter. In order to chronically pace freely roaming mice, we integrated a transmitter coil into the mouse housing. This system is designed to generate voltage fields in excess of the 3.9 V cap on the receiver throughout the cage to eliminate communication issues between the transmitter and receiver. This will ensure that mice will always be paced independent of the location they occupy in the cage.

The development of a practical pacemaker for long-term studies in the mouse will permit a range of future studies. First, the development of the mouse as a model of cardiac memory will allow the well-developed molecular and genetic tools available in this animal to be brought to bear on the mechanism(s) underlying pacemaker-induced cardiac electrical remodeling. Second, long-term pacing at supra-physiological rates can result in heart failure and atrial fibrillation, both common pathologies amenable to further genetic studies in the mouse. While the current design is sufficient for physiologic testing, future development will focus on potential improvement to the pacing electrode and the creation of an electrical sensing circuit. During some experiments, subtle chest-wall captured was observed during pacing. Further modifications of the lead design with novel printing strategies on flexible substrates and improvements to the surgical method should mitigate this issue. Additionally, our receiver circuit is a dedicated pacing circuit without sensing capabilities. Stimulation and sensing with radiofrequency induction has been demonstrated previously for neural applications [12]. Future research will focus on adopting this technology for our pacemaker. We anticipate that further development of this technology will open the door to a range of cardiac studies in the mouse.

## Supporting Information

File S1 **Source code used in the first generation of implantable pacemaker.**
(DOCX)
