# Supplementary material for: A Fully Implantable Pacemaker for the Mouse: From Battery to Wireless Power
Source: PLoS One. 2013 Oct 23;8(10):e76291. doi: 10.1371/journal.pone.0076291 (PMC3806780; doi:10.1371/journal.pone.0076291)
Supplement: File S1 — Source code used in the first generation of implantable pacemaker. (DOCX) [file pone.0076291.s001.docx]

Supplemental Source Code for Battery-Powered Pacemaker

;===================================================================

;

; MousePacemaker_HigherLevel_SourceCode.asm

;

; Washington University in St. Louis

;

; Efimov Lab

;

; using the PIC12F675

; Date: July 13, 2010

; Clock frequency: 4MHz

;

; This is only a draft of the code for pacemaker,

; will be debugged and added more functions in the future.

;

;===================================================================

list p=12f675

#include "P12F675.INC"

;Config:

__CONFIG _MCLRE_ON & _LP_OSC & _CP_OFF & _CPD_OFF & _WDT_OFF & _BODEN_OFF & _PWRTE_OFF

;===================================================================

;Defines

;===================================================================

#define TXD GPIO,0 ;define output port GP0

#define SW1 GPIO,2 ;define interrupt port GP2 --> SW1

#define Count1 0x20 ;define data bank1

;===================================================================

;Reset Vector

;===================================================================

ORG 0x000 ; processor reset vector

goto Start ; go to beginning of program

ORG 0x004 ;Interrupt vector location

goto INT_SERV

;------------- VARS : variables into file register bank--------------

cblock 0x21 ; (bank 0 0x21-0x5f are free for use)

d1

d2

d3

endc

;--------------Initialization ROUTINE-----------------------------------------

Init

clrwdt

BSF STATUS,RP0 ; bank 1

movlw b'11111111' ; enable all WPU

movwf WPU

movlw b'01111000' ; enable global pull-ups

movwf OPTION_REG

movlw 04h ; GP1:2 interrupt pins

movwf IOC

movlw b'111110' ; GP0 output & GP1:5 Input

movwf TRISIO

clrf PIE1 ; turn off peripheral ints

clrf ANSEL ; digital GPIO only, no analog

clrf VRCON ; Vreff off

BCF STATUS,RP0 ; go to Bank 0

clrf GPIO ; Init GPIO

movlw 07h ; GP<0:2> to digital IO

movwf CMCON ; comparator off

clrf T1CON ; turn off TMR1

clrf ADCON0 ; turn off A to D conv.

CLRF Count1 ; zero the counter

bcf TXD ; no voltage discharge for initial stage

movlw b'11001000' ; Enable GPIO Interrupt,

movwf INTCON ; and enables global interrupts

;define initial counter values

movlw d'15'

movwf Count1

goto Routine

;===================================================================

;Interrupt

;===================================================================

INT_SERV:

;-------SW1 Check-------

Wait

btfss SW1 ;wait for button release/debounce

goto Wait ;wait if not interrupt

DECFSZ Count1,f

goto CleaerFlag

goto Reset_Count1

Reset_Count1

movlw d'15'

movwf Count1

goto CleaerFlag

CleaerFlag

movlw b'11001000' ; Enable GPIO Interrupt,

movwf INTCON

GOTO Stage#

RETFIE

;--------------MAIN ROUTINE-----------------------------------------

Start

goto Init

Routine

goto Stage#

Main

decfsz d1, f

goto $+2

decfsz d2, f

goto Main

goto Routine ;loop back

;-------Stages-------

;Stage 15 Sleep (Off)

;Stage 14 "frequency=600; PD=2.5ms"

;Stage 13 "frequency=7000; PD=2.5ms"

;Stage 12 "frequency=800; PD=2.5ms"

;Stage 11 "frequency=1000; PD=2.5ms"

;Stage 10 "frequency=600; PD=2ms"

;Stage 9 "frequency=700; PD=2ms"

;Stage 8 "frequency=800; PD=2ms"

;Stage 7 "frequency=1000; PD=2ms"

;Stage 6 "frequency=600; PD=3ms"

;Stage 5 "frequency=700; PD=3ms"

;Stage 4 "frequency=800; PD=3ms"

;Stage 3 "frequency=1000; PD=3ms"

;Stage 2 "frequency=800; PD=5ms"

;Stage 1 "frequency=1000; PD=5ms"

;Stage 0 _______________

;****************************************************************************

;Binary search routine to check "Count1" from SW1

;****************************************************************************

Stage#

PDXXXX BTFSS Count1, 3 ; Test the first bit. Skip the next line if the bit equals 1

GOTO PD0XXX ; Go to the next test, knowing that the first bit is zero.

GOTO PD1XXX ; Go to the next test, knowing that the first bit is one.

PD0XXX BTFSS Count1, 2 ; Test the second bit. Skip the next line if the bit equals 1.

GOTO PD00XX ; Go to the next test, knowing that the second bit is zero.

GOTO PD01XX ; Go to the next test, knowing that the second bit is one.

PD1XXX BTFSS Count1, 2 ; Test the second bit. Skip the next line if the bit equals 1.

GOTO PD10XX ; Go to the next test, knowing that the second bit is zero.

GOTO PD11XX ; Go to the next test, knowing that the second bit is one.

PD00XX BTFSS Count1, 1 ; Test the third bit. Skip the next line if the bit equals 1.

GOTO PD000X

GOTO PD001X

PD01XX BTFSS Count1, 1 ; Test the third bit. Skip the next line if the bit equals 1.

GOTO PD010X

GOTO PD011X

PD10XX BTFSS Count1, 1 ; Test the third bit. Skip the next line if the bit equals 1.

GOTO PD100X

GOTO PD101X

PD11XX BTFSS Count1, 1 ; Test the third bit. Skip the next line if the bit equals 1.

GOTO PD110X

GOTO PD111X

PD000X BTFSS Count1, 0 ; Test the fourth bit. Skip the next line if the bit equals 1

GOTO PD0000

GOTO PD0001

PD001X BTFSS Count1, 0 ; Test the fourth bit. Skip the next line if the bit equals 1.

GOTO PD0010

GOTO PD0011

PD010X BTFSS Count1, 0 ; Test the fourth bit. Skip the next line if the bit equals 1.

GOTO PD0100

GOTO PD0101

PD011X BTFSS Count1, 0 ; Test the fourth bit. Skip the next line if the bit equals 1.

GOTO PD0110

GOTO PD0111

PD100X BTFSS Count1, 0 ; Test the fourth bit. Skip the next line if the bit equals 1.

GOTO PD1000

GOTO PD1001

PD101X BTFSS Count1, 0 ; Test the fourth bit. Skip the next line if the bit equals 1.

GOTO PD1010

GOTO PD1011

PD110X BTFSS Count1, 0 ; Test the fourth bit. Skip the next line if the bit equals 1.

GOTO PD1100

GOTO PD1101

PD111X BTFSS Count1, 0 ; Test the fourth bit. Skip the next line if the bit equals 1.

GOTO PD1110

GOTO PD1111

;***************************************************************************************

;***************************************************************************************

PD1111 ;15

bcf TXD ;Output off

sleep ;sleep/off state

nop

goto INT_SERV

PD1110 ;14

BSF GPIO,0 ;turn on GPIO,0

goto $+1 ;2040uS

goto $+1

goto $+1

goto $+1

goto $+1

goto $+1

goto $+1

goto $+1

BCF GPIO,0

movlw 0x9a ;600BPM

movwf d1

movlw 0x01

movwf d2

goto Main

PD1101 ;13

BSF GPIO,0 ;turn on GPIO,0

goto $+1 ;2040uS

goto $+1

goto $+1

goto $+1

goto $+1

goto $+1

goto $+1

goto $+1

BCF GPIO,0

movlw 0x83 ;700BPM

movwf d1

movlw 0x01

movwf d2

goto Main

PD1100 ;12

BSF GPIO,0 ;turn on GPIO,0

goto $+1 ;2040uS

goto $+1

goto $+1

goto $+1

goto $+1

goto $+1

goto $+1

goto $+1

BCF GPIO,0

movlw 0x72 ;800BPM

movwf d1

movlw 0x01

movwf d2

goto Main

PD1011 ;11

BSF GPIO,0 ;turn on GPIO,0

goto $+1 ;2040uS

goto $+1

goto $+1

goto $+1

goto $+1

goto $+1

goto $+1

goto $+1

BCF GPIO,0

movlw 0x5a ;1000BPM

movwf d1

movlw 0x01

movwf d2

goto Main

PD1010 ;10

BSF GPIO,0 ;turn on GPIO,0

goto $+1 ;25200uS

goto $+1

goto $+1

goto $+1

goto $+1

goto $+1

goto $+1

goto $+1

goto $+1

goto $+1

BCF GPIO,0

movlw 0x97 ;600BPM

movwf d1

movlw 0x01

movwf d2

goto Main

PD1001 ;9

BSF GPIO,0 ;turn on GPIO,0

goto $+1 ;25200uS

goto $+1

goto $+1

goto $+1

goto $+1

goto $+1

goto $+1

goto $+1

goto $+1

goto $+1

BCF GPIO,0

movlw 0x80 ;700BPM

movwf d1

movlw 0x01

movwf d2

goto Main

PD1000 ;8

BSF GPIO,0 ;turn on GPIO,0

goto $+1 ;25200uS

goto $+1

goto $+1

goto $+1

goto $+1

goto $+1

goto $+1

goto $+1

goto $+1

goto $+1

BCF GPIO,0

movlw 0x72 ;800BPM

movwf d1

movlw 0x01

movwf d2

goto Main

PD0111 ;7

BSF GPIO,0 ;turn on GPIO,0

goto $+1 ;25200uS

goto $+1

goto $+1

goto $+1

goto $+1

goto $+1

goto $+1

goto $+1

goto $+1

goto $+1

BCF GPIO,0

movlw 0x59 ;1000BPM

movwf d1

movlw 0x01

movwf d2

goto Main

PD0110 ;6

BSF GPIO,0 ;turn on GPIO,0

goto $+1 ;3120uS

goto $+1

goto $+1

goto $+1

goto $+1

goto $+1

goto $+1

goto $+1

goto $+1

goto $+1

goto $+1

goto $+1

BCF GPIO,0

movlw 0x97 ;600BPM

movwf d1

movlw 0x01

movwf d2

goto Main

PD0101 ;5

BSF GPIO,0 ;turn on GPIO,0

goto $+1 ;3120uS

goto $+1

goto $+1

goto $+1

goto $+1

goto $+1

goto $+1

goto $+1

goto $+1

goto $+1

goto $+1

goto $+1

BCF GPIO,0

movlw 0x7f ;700BPM

movwf d1

movlw 0x01

movwf d2

goto Main

PD0100 ;4

BSF GPIO,0 ;turn on GPIO,0

goto $+1 ;3120uS

goto $+1

goto $+1

goto $+1

goto $+1

goto $+1

goto $+1

goto $+1

goto $+1

goto $+1

goto $+1

goto $+1

BCF GPIO,0

movlw 0x6f ;800BPM

movwf d1

movlw 0x01

movwf d2

goto Main

PD0011 ;3

BSF GPIO,0 ;turn on GPIO,0

goto $+1 ;3120uS

goto $+1

goto $+1

goto $+1

goto $+1

goto $+1

goto $+1

goto $+1

goto $+1

goto $+1

goto $+1

goto $+1

BCF GPIO,0

movlw 0x57 ;1000BPM

movwf d1

movlw 0x01

movwf d2

goto Main

PD0010 ;2

BSF GPIO,0 ;turn on GPIO,0

goto $+1 ;5160uS

goto $+1

goto $+1

goto $+1

goto $+1

goto $+1

goto $+1

goto $+1

goto $+1

goto $+1

goto $+1

goto $+1

goto $+1

goto $+1

goto $+1

goto $+1

goto $+1

goto $+1

goto $+1

goto $+1

BCF GPIO,0

movlw 0x6b ;800BPM

movwf d1

movlw 0x01

movwf d2

goto Main

PD0001 ;1

BSF GPIO,0 ;turn on GPIO,0

goto $+1 ;5160uS

goto $+1

goto $+1

goto $+1

goto $+1

goto $+1

goto $+1

goto $+1

goto $+1

goto $+1

goto $+1

goto $+1

goto $+1

goto $+1

goto $+1

goto $+1

goto $+1

goto $+1

goto $+1

goto $+1

BCF GPIO,0

movlw 0x53 ;1000BPM

movwf d1

movlw 0x01

movwf d2

goto Main

PD0000 ;0

goto Main

End
